# Phylogeny of Chinese *Allium* Species in Section *Daghestanica* and Adaptive Evolution of *Allium* (Amaryllidaceae, Allioideae) Species Revealed by the Chloroplast Complete Genome

**DOI:** 10.3389/fpls.2019.00460

**Published:** 2019-04-30

**Authors:** Deng-Feng Xie, Huan-Xi Yu, Megan Price, Chuan Xie, Yi-Qi Deng, Jun-Pei Chen, Yan Yu, Song-Dong Zhou, Xing-Jin He

**Affiliations:** ^1^Key Laboratory of Bio-Resources and Eco-Environment of Ministry of Education, College of Life Sciences, Sichuan University, Chengdu, China; ^2^Sichuan Key Laboratory of Conservation Biology on Endangered Wildlife, College of Life Sciences, Sichuan University, Chengdu, China

**Keywords:** *Allium*, Allioideae, chloroplast genome, phylogeny analyses, adaptive evolution, positive selection

## Abstract

The genus *Allium* (Amaryllidaceae, Allioideae) is one of the largest monocotyledonous genera and it includes many economically important crops that are cultivated for consumption or medicinal uses. Recent advances in molecular phylogenetics have revolutionized our understanding of *Allium* taxonomy and evolution. However, the phylogenetic relationships in some *Allium* sections (such as the *Allium* section *Daghestanica*) and the genetic bases of adaptative evolution, remain poorly understood. Here, we newly assembled six chloroplast genomes from Chinese endemic species in *Allium* section *Daghestanica* and by combining these genomes with another 35 allied species, we performed a series of analyses including genome structure, GC content, species pairwise Ka/Ks ratios, and the SSR component, nucleotide diversity and codon usage. Positively selected genes (PSGs) were detected in the *Allium* lineage using the branch-site model. Comparison analysis of Bayesian and ML phylogeny on CCG (complete chloroplast genome), SCG (single copy genes) and CDS (coding DNA sequences) produced a well-resolved phylogeny of Allioideae plastid lineages, which illustrated several novel relationships with the section *Daghestanica*. In addition, six species in section *Daghestanica* showed highly conserved structures. The GC content and the GC3s content in Allioideae species exhibited lower values than studied non-Allioideae species, along with elevated pairwise Ka/Ks ratios. The *rps2* gene was lost in all examined Allioideae species, and 10 genes with significant posterior probabilities for codon sites were identified in the positive selection analysis, seven of them are associated with photosynthesis. Our study uncovered a new species relationship in section *Daghestanica* and suggested that the selective pressure has played an important role in *Allium* adaptation and evolution, these results will facilitate our further understanding of evolution and adaptation of species in the genus *Allium*.

## Introduction

**FIGURE 1:**
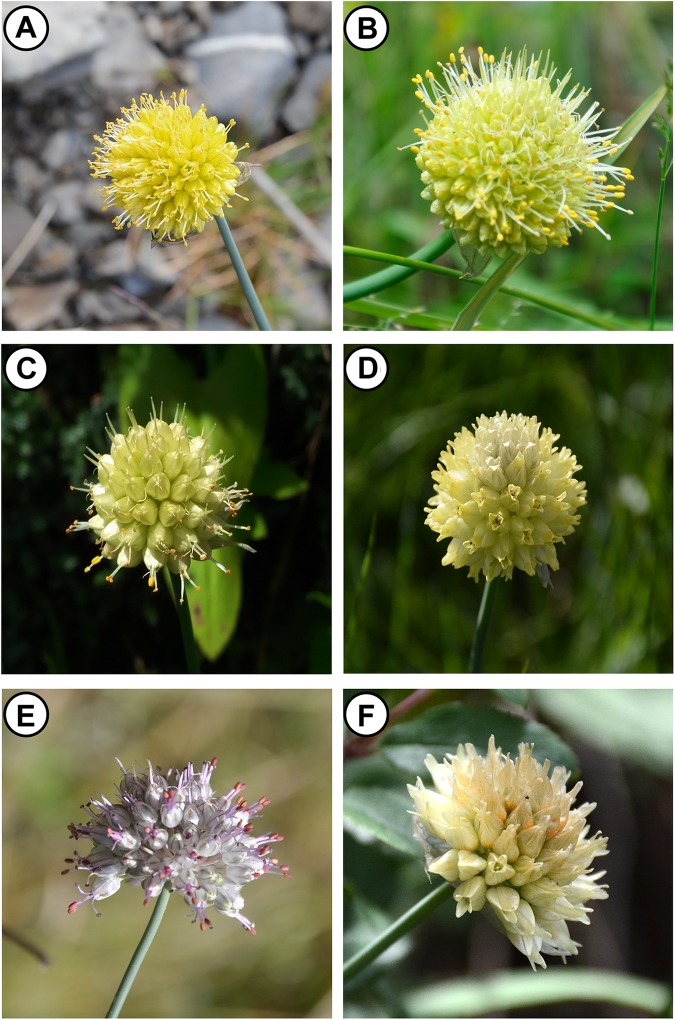
The flower morphological characters of Section *Daghestanica* species. **(A)** *A. chrysanthum*, **(B)** *A. rude*, **(C)** *A. xichuanense*, **(D)** *A. chrysocephalum*, **(E)** *A. maowenense*, and **(F)** *A. herderianum*.

*Allium* L. is the single genus of Allieae and belongs to the subfamily Allioideae (Amaryllidaceae) as per update APG IV (Chase et al., 2016). It is one of the largest genera of the monocotyledons (∼ 920 species) and includes many economically important crops (Herden et al., 2016; Zhu et al., 2017). The new classification of *Allium* was made by Friesen et al. (2006), who first suggested that the genus *Allium* is monophyletic. Despite extensive work on the genus, taxonomical and phylogenetic uncertainties remain in some subgenera or sections. For example, *Allium* section *Daghestanica* (Tscholok.) N. Friesen. has recently been proposed to be a small group (Friesen et al., 2006) containing more than 10 species globally, with six being endemic to China according to Li et al. (2010). The six Chinese endemics are primarily distributed in the southeast fringe of the Qinghai-Tibet Plateau (QTP): *A. chrysanthum* Regel, *A. chrysocephalum* Regel, *A. herderianum* Regel, *A. rude* J.M.Xu, *A. xichuanense* J.M.Xu, and *A. maowenense* J.M.Xu (Figure 1). Early studies placed *A. rude*, *A. xichuanense*, *A. chrysocephalum* and *A. herderianum* into sect. *Rhiziridium* G. Don, *A. chrysanthum* was placed into sect. *Schoenoprasum* G. Don, and the *A. maowenense* was classified into sect. *Haplostemon* Boiss (Xu et al., 1994; Chen et al., 2000). Li et al. (2010) then reclassified the species into sect. *Daghestanica* according to molecular phylogenetic analyses, and was confirmed by morphological evidences produced by Yu et al. (2018). These previous studies have significantly advanced the phylogeny and taxonomy of the six species, yet a consensus of the six species’ exact relationships have not been reached. In particular, uncertainties remain because *A. herderianum* was not sampled in previous phylogenetic studies (Li et al., 2010).

Many nuclear genes and chloroplast genomes were recently employed in *Allium* studies (Friesen et al., 2006; Nguyen et al., 2008; Kim and Yoon, 2010; Li et al., 2010; Lee et al., 2017; Jin et al., 2018), which provide valuable information for the phylogenetic study of section *Daghestanica*. In particular, the whole chloroplast genomes that possess highly conserved gene structure and gene content, and lower substitution rate than nuclear DNA (especially in inverted repeat regions), offer promising solutions to phylogeny uncertainties (Wolfe et al., 1987; Raubeson et al., 2005; Parks et al., 2009). The chloroplast genome sequence of *A. cepa* is such a single circular molecule of 153,440 bp length with a quadripartite structure (containing 132 genes) that includes two copies: LSC (large single-copy) and SSC (small single-copy), which are separated by an IR (inverted repeats) region (Kim et al., 2015).

In addition to their applicability for phylogenetic studies, whole chloroplast genomes can provide insights into other evolutionary processes, such as chloroplast inheritance, domestication studies and adaptive evolution. Adaptive evolution, defined as the adaptability improvement of species during their evolutionary processes, and it is always driven by evolutionary processes such as natural selection, which act on genetic variations produced by mutations, genetic recombination and gene flow (Scottphillips et al., 2014) and resulted in biodiversity at every level of biological organization (Hall et al., 2008). Therefore, selection pressures that species experienced in their evolutionary processes constitute another interesting aspect in chloroplast genomes analyses. Recent studies detected many positively selected chloroplast genes [genes with Ka (non-synonymous substitution) greater than Ks (synonymous substitutions)]. For example, *rbcL* and nuclear *rbcS* genes in *Flaveria* (Kapralov et al., 2011), as well as *clpP1* exon in three distantly related taxa of *Oenothera* (Erixon and Oxelman, 2008).

Species of *Allium* are all perennial herbs, are distributed widely from the dry subtropics to the boreal zones, and are characterized by diversified morphological features (e.g., bulbs, leaves, and flowers). Furthermore, the habitat of *Allium* species varies from dry and well-drained soils to moist and organic soils, that can be in swamps or water (Block, 2010). Therefore, *Allium* is considered as a successful taxon due to its wide distribution and diversification (Li et al., 2010). Generally, substitution rates of angiosperm cp genomes are slow and are minimally affected by adaptive evolution (Erixon and Oxelman, 2008), excluding several genes that may evolve very rapidly due to the effects of positive selection (Ivanova et al., 2017). Previous studies have found that the positive selection is expected to accelerate the Ka value yet it does not affect the Ks value (Fan et al., 2018). However, little is known about the positive selection and adaptation of *Allium* species.

In this study, the whole cp genomes of Chinese species in section *Daghestanica* were sequenced using the next-generation sequencing platform. Combined with another 35 cp genomes previously published (including species from Asparagaceae, Allioideae, Agapanthoideae, Asphodeloideae, Asphodelaceae, and Iridaceae), here we provide the comprehensive analysis of cp genomes for *Allium* and species in allied families based on present cp genome data. We aimed to generate a robust phylogeny of extant *Allium* cp genome data, and used this phylogeny to: (1) reconstruct the phylogeny of Chinese species in *Allium* section *Daghestanica* based on the cp genome data and analyze the species relationships at the plastid level. (2) compare the cp genome structure of species within section *Daghestanica*, in genus *Allium* (Allioideae) and other allied families; and (3) investigate selective or adaptive evolution in the cp genomes of *Allium* species.

## Materials and Methods

### Plant Materials and DNA Extraction

We collected the fresh leaves from each field site (Supplementary Table S1) and were immediately used for DNA extraction. The total genomic DNA was extracted from leaf tissues with a modified Cetyl Trimethyl Ammonium Bromide (CTAB) method (Doyle, 1987).

### Plastome Genome Sequencing and Assembling

All genome data were sequenced using an Illumina Hiseq 2500 platform by Biomarker Technologies, Inc (Beijing, China). High-quality reads were obtained using the CLC Genomics Workbench v7.5 (CLC Bio, Aarhus, Denmark). Reference-guided assembly was then performed to reconstruct the chloroplast genomes using the program MITObim v1.7 (Christoph et al., 2013). In order to obtain accurate sequences, each species was assembled four times with the reference genomes *A. cepa* (KM088014), *A. sativum* (KY085913), *A. victorialis* (NC_037240), and *A. obliquum* (LT699701). Gaps that appeared in the assembled cp genomes were corrected by Sanger sequencing and the primers were designed using Lasergene 7.1 (DNASTAR, Madison, WI, United States). The primers and amplifications were shown in Supplementary Table S2. The program DOGMA (Wyman et al., 2004) was used to annotate the whole cp genome, and subsequently corrected within GENEIOUS R11 (Biomatters, Ltd., Auckland, New Zealand). Final plastid genome maps were drawn using OGDRAW (Lohse et al., 2013).

### GC Content and Species Pairwise Ka/Ks Ratios

GC content of the complete chloroplast genome (CCG) and the third position GC content of codons in each species were calculated using PAML v4.8 (Yang, 2007). Each CDS sequence of all species was extracted and aligned with MAFFT v. 7 (Katoh and Standley, 2013). Pairwise Ka/Ks ratios of all species were calculated using the concatenated single-CDS alignments with KaKs Calculator version 2.0 (Wang et al., 2010).

### SSRs Characterization and Chloroplast Genome Nucleotide Diversity

Perl script MISA (Thiel et al., 2003) was used to search microsatellites loci in the cp genomes with parameters being set as 10, 5, 4, 3, 3, and 3 for mono-, di-, tri-, tetra-, penta-, and hexa-nucleotides, respectively. The DnaSP version 5.1 (Librado and Rozas, 2009) was used to calculate the nucleotide diversity of genes in LSC, SSC, and IR regions.

### Indices of Codon Usage

Codon usage in these genes was assessed using the program codon W 1.4.4 (J. Peden)^1^. Five values were used to estimate the extent of bias toward codons: the codon adaptation index (CAI), codon bias index (CBI), frequency of optimal codons (Fop), GC content of synonymous third codons positions (GC3s), and the effective number of codons (ENC).

### Phylogenetic Analyses

In order to investigate the relationships of the six *Allium* species, all available complete genome sequences in allied families were downloaded from NCBI, including 20 species from Asparagaceae, 10 species in Allioideae (all *Allium* species), one species in Agapanthoideae, 2 species in Asphodeloideae, one species in Asphodelaceae and one species in Iridaceae (Supplementary Table S3). Firstly, all single-copy genes were extracted from all 41 taxa, and alignments of each gene were generated and trimmed. These alignments were then concatenated to produce an alignment of all single copy genes, which were used for phylogenetic analysis. Maximum likelihood (ML) analyses were performed using RAxML 8.2.8 (Stamatakis, 2014) with GTR + G model and 1,000 bootstrap replicates. Bayesian analyses were performed with MrBayes v. 3.2.5 (Ronquist and Huelsenbeck, 2003) under the GTR + I + Γ substitution model. The Markov chain Monte Carlo (MCMC) algorithm was run for 1 × 10^8^ generations, with one tree sampled every 1000 generations. The first 20% of trees were discarded as burn-in, and the remaining trees were used to build a 50% majority-rule consensus tree. The stationarity was considered to be reached when the average standard deviation of split frequencies remained below 0.001. In view of the utility of different cp regions, phylogenetic analyses were performed for the CCG data and the CDS sequences respectively.

### Positive Selected Analyses

An optimized branch-site model (Yang and Dos, 2011) and Bayesian Empirical Bayes (BEB) methods (Yang et al., 2005) were used to identify genes under positive selection in *Allium* species (Allioideae) compared to species in non-Allioideae families. The single-copy CDS sequences of all 41 taxa were extracted and the software MUSCLE v3.6 (Edgar, 2004) was used in sequence alignment according to their amino acid sequences. The alignments of the DNA codon sequences were further trimmed by TRIMAL v1.2 (Capellagutiérrez et al., 2009), and the final alignments were used to perform the positive selection analyses. The branch-site model was implemented to assess potential positive selection in specifically designated Allioideae lineage in the PAML v4.8 package (Yang, 2007). The ratio (ω) of the non-synonymous substitution rate (Ka) to the synonymous substitutions rate (Ks) was used to measure the selective pressure. The ratio ω > 1, ω = 1, and ω < 1 suggest positive selection, neutral selection and negative selection, respectively (Yang and Nielsen, 2002). The log-likelihood values were calculated and tested according to Lan et al. (2017). The BEB method was applied to compute the posterior probabilities of amino acid sites to identify whether these specific sites were under positive selection (codon sites with a high posterior probability) (Yang et al., 2005). A gene with a test *p*-value < 0.05 and with positively selected sites was considered as a positively selected gene (PSG). The Jalview v2.4 (Clamp et al., 2004) was used to view the amino acid sequences of PSGs.

## Results

### Chloroplast Features of *Allium* Species

**FIGURE 2:**
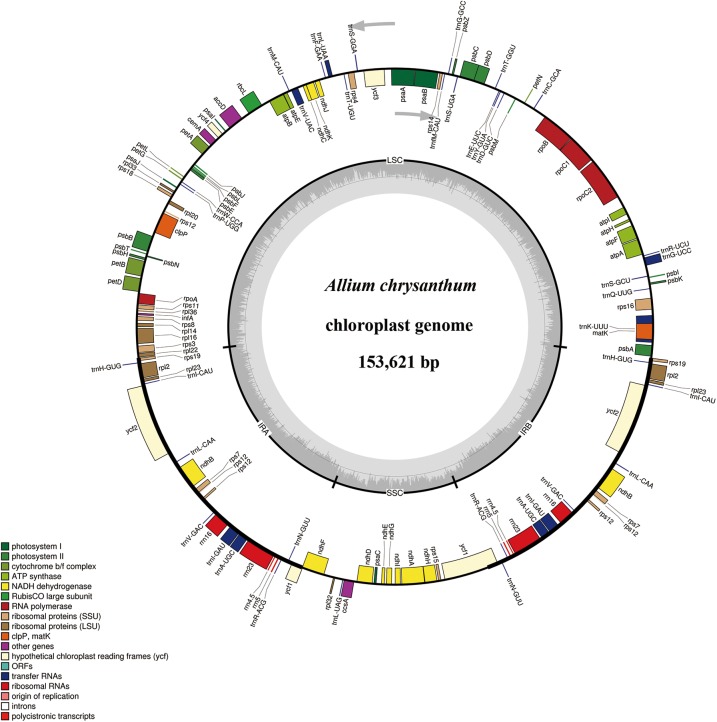
Gene maps of the Section *Daghestanica* species chloroplast (cp) genomes. Genes shown inside the circle are transcribed clockwise, and those outside are transcribed counterclockwise. Genes belonging to different functional groups are color-coded. The darker gray color in the inner circle corresponds to the GC content, and the lighter gray color corresponds to the AT content. SSU, small subunit; LSU, large subunit; ORF, open reading frame.

The complete cp genomes of six *Allium* species ranged from 153,605 bp (*A. herderianum*) to 153,710 bp (*A. chrysocephalum*) in length, with the minimum and maximum differences being 3 and 105 bp, respectively (Table 1 and Figure 2). All six cp genomes showed a typical quadripartite structure that was similar to those of most land plants. The cp genome consisted of a pair of IR regions (26,446–26,512 bp) separated by the LSC (82,658–82,815 bp) and SSC (17,950–18,000 bp) regions. The GC content ranged from 37.7–37.8%, indicating nearly identical levels among the six *Allium* cp genomes. In addition, the six *Allium* cp genomes encoded 132 functional genes, with 86 protein-coding genes, 38 tRNA genes, and eight ribosomal RNA genes (Table 1 and Supplementary Table S4). The length and GC contents of the non-coding regions in the six *Allium* species were lower than the whole cp genome and the coding regions (Table 1). The length, GC content and gene components of the 41 species were included in the Supplementary Table S5.

**Table 1:** Summary of complete chloroplast genomes of Section *Daghestanica* species.

Taxon	Full		LSC length (bp)	SSC length (bp)	IR length (bp)	Gene Number	Protein-coding	tRNAs	rRNAs	Coding region		Noncoding region	
									
	length (bp)	GC (%)								length (bp)	GC (%)	length (bp)	GC (%)
*Allium chrysanthum*	153,621	37.8	82744	17985	26446	132	86	38	8	79269	37.2	74352	36.37
*Allium rude*	153,697	37.7	82815	17978	26452	132	86	38	8	79284	37.2	74413	36.17
*Allium xichuanense*	153,673	37.8	82797	17950	26463	132	86	38	8	79269	37.2	74404	36.17
*Allium chrysocephalum*	153,710	37.8	82688	17998	26512	132	86	38	8	79280	37.2	74430	36.37
*Allium maowenense*	153,608	37.8	82668	18000	26470	132	86	38	8	79256	37.2	74352	36.37
*Allium herderianum*	153,605	37.8	82658	17983	26482	132	86	38	8	79276	37.2	74329	36.37


*LSC, large single-copy; SSC, small single-copy; IR, inverted repeat.*

### GC Content Distribution and the Ka/Ks Ratios of Species Pairwise

**FIGURE 3:**
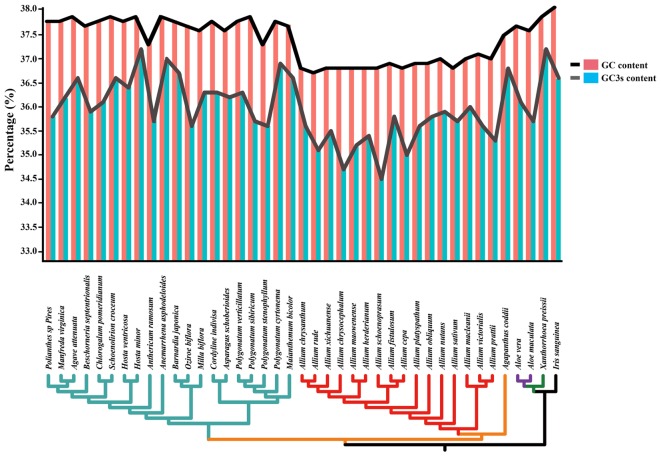
Changes in plastid GC content of all 41 taxa. This graph shows the total GC content (red bar and black line) and the third codon position GC content (blue bar and gray line) of each species.

The total and the third position GC content were compared between 41 species (belonging to Asparagaceae, Allioideae, Agapanthoideae, Asphodeloideae, Asphodelaceae, and Iridaceae). Lower GC contents were observed at the total nucleotides level (<38.5%) and the third codon positions (<36.0%) in most of *Allium* (Allioideae) species compared to species in non-Allioideae families (Figure 3 and Supplementary Table S6).

**FIGURE 4:**
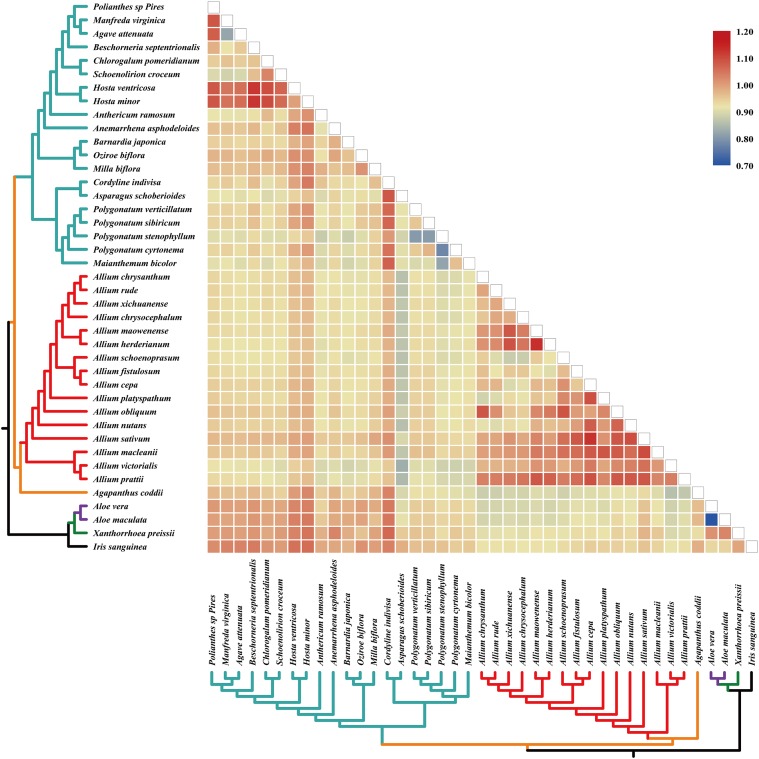
Pairwise Ka/Ks ratios in *Allium* (Allioideae) and other families. This heatmap shows pairwise Ka/Ks ratios between every sequence in the multigene nucleotide alignment. *Allium* (Allioideae) are shown on red branches. The scale factors associated with each value are shown on the right-hand side of the figure.

The pairwise Ka/Ks ratios of each species pair were calculated (Figure 4), which provided information of selective pressure that acted on individual sequences. Much higher pairwise Ka/Ks ratios were observed in *Allium* (Allioideae) species pairs than non-Allioiseae species pairs (Figure 4 and Supplementary Table S7). In addition, high Ka/Ks ratios were also detected in other species (e.g., species in *Hosta* and *Cordyline*) (Figure 4 and Supplementary Table S7).

### Repeat Sequences Variations, the Nucleotide Diversity, Codon Usage and Gene Loss

We detected numerous microsatellites (SSRs) in the six *Allium* cp genomes, ranging from 179 (*A. maowenense*) to 193 (*A. chrysocephalum*) (Supplementary Figure S1). The most abundant were mono-nucleotide repeats, where the number varied from 63 in *A. chrysocephalum* to 74 in *A. rude*, followed by tetra-nucleotides and di-nucleotide repeats, while the penta-nucleotide repeats were the least abundant (Supplementary Figure S1 and Supplementary Table S8). The overall length of the five categories of perfect SSRs ranged from 9 to 25 bp in the six *Allium* species (Supplementary Table S8).

The nucleotide diversity values in the LSC regions ranged from 0.0006 to 0.07823 with a mean value of 0.0310 (from 0.0035 to 0.0722 with the average value was 0.0465 in SSC regions), while the value was from 0.0000 to 0.0311 with a mean value of 0.0084 in the IRs regions (Supplementary Figure S2). Six genes with high nucleotide diversity were detected (>0.0700), these were *trnK-UUU*, *matK*, *trnG-UCC*, *trnG-GCC*, *ndhF* and *rps15*. Five genes (i.e., *accD*, *clpP*, *rpl16*, *ccsA* and *ndhA*) with nucleotide diversity more than 0.05500 were also detected.

**FIGURE 5:**
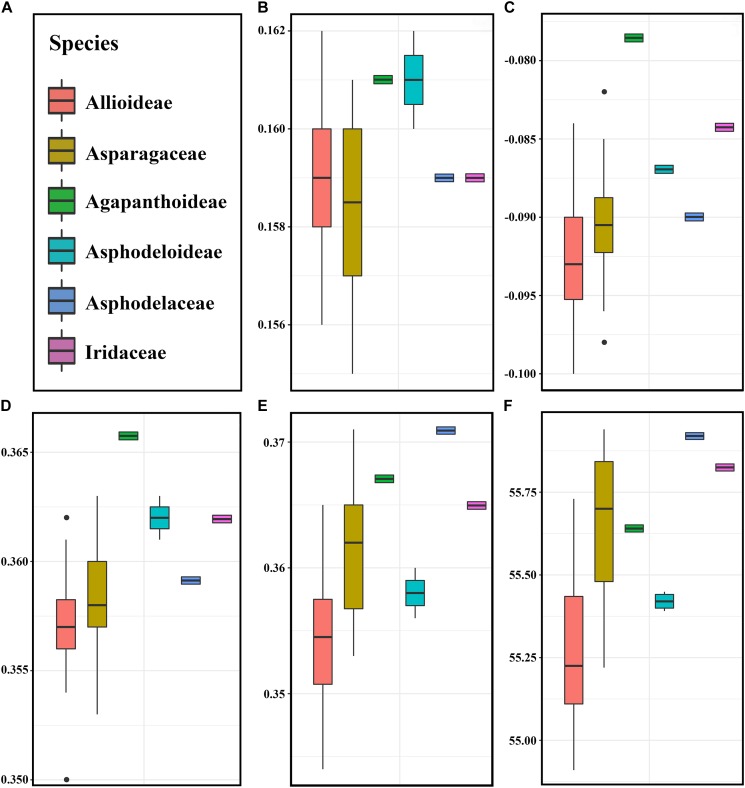
The comparative analysis of codon usage bias in *Allium* (Allioideae) and other family species. **(A)** Labels representing each of families. **(B)** CAI (Codon adaptation index), **(C)** CBI (Codon bias index), **(D)** FOP (Frequency of optimal codons index), **(E)** GC3s (GC of synonymous codons in 3rd position), **(F)** ENC (Effective number of codons).

The pattern of codon usage bias in the *Allium* (Allioideae) and non-Allioideae were investigated. We found that five parameters involved in codon usage bias were lower in *Allium* (Allioideae) species than non-Allioideae species, except the CAI that was lower in the family Asparagaceae (Figure 5).

**FIGURE 6:**
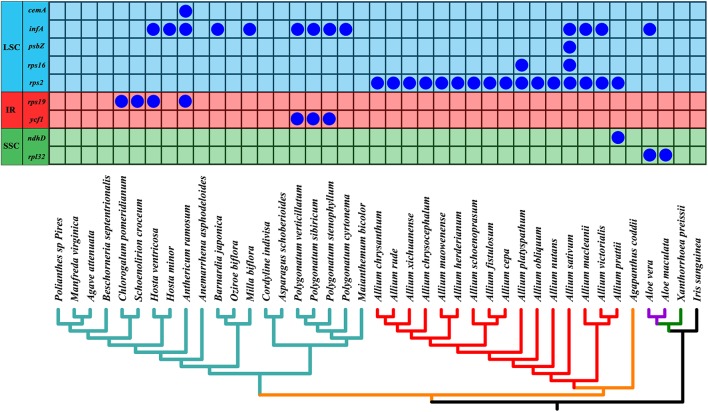
Loss of chloroplast protein-coding genes in the phylogeny of all 41 taxa. Below is the phylogeny of all 41 species based on chloroplast genomes as shown in Figure 7. Different chloroplast regions were indicated at the left side. Allioideae are shown on red branches. IR, inverted repeat; LSC, large single-copy region; SSC, small single-copy region.

As shown in Figure 6, we found that the gene *rps2* was lost in all *Allium* (Allioideae) species. In addition, four genes *infA*, *rps16*, *psbZ*, and *ndhD* were lost in four *Allium* species with different degree (*A. sativum*, *A. macleanii*, *A. platyspathum*, and *A. victorialis*). Gene *cemA*, *infA, rps19, and ycf1* were lost in some species of Asparagaceae, and gene *rpl32* and *infA* were missing in *Aloe vera* and *A. maculate* of Asphodeloideae.

### Characteristics of cp Genome and Phylogenetic Analysis

Complete chloroplast genome of the six species in section *Daghestanica* were newly sequenced in this study, and were deposited in GenBank (Supplementary Table S3). These plastid genomes are similar to previously published *Allium* plastomes in size, structure and gene content (Filyushin et al., 2016, 2018; Lee et al., 2017; Jin et al., 2018). The CCG data set had an aligned length of 192056 bp, within which 24727 parsimony-informative sites (PICs, 12.87 %) were detected. The SCG (single copy genes) possessed 54401 bp aligned nucleotides with 6755 PICs (12.41 %). CDS (coding DNA sequences) data set had an aligned length of 45954 bp nucleotides with 5464 PICs (11.89 %). Comparing these data sets with CCG, the percentages of PICs, SCG and CDS were reduced.

**FIGURE 7:**
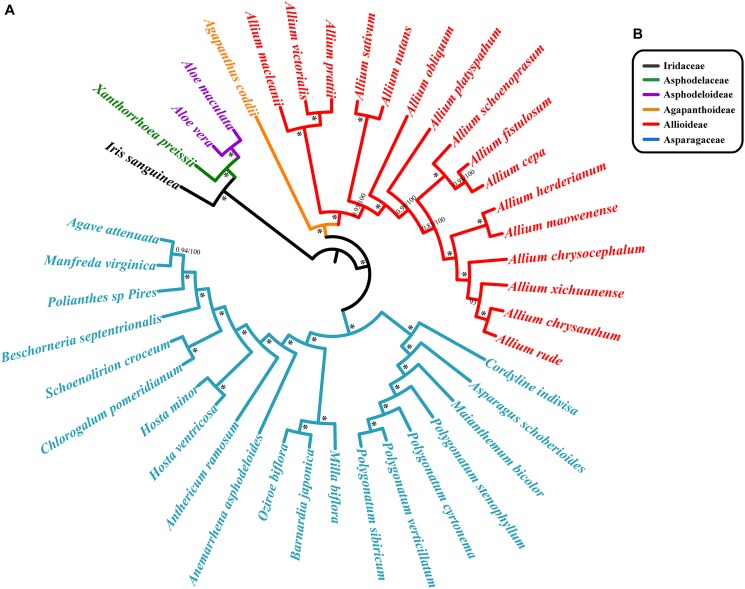
Phylogenetic relationships of section *Daghestanica* species with related 35 species based on the whole cp genomes. **(A)** Tree constructed by Bayesian inference (BI) and maximum likelihood (ML) with the posterior probabilities of BI and the bootstrap values of ML above the branches, respectively. **^∗^**Represent maximum support in all two analyses. **(B)** Families of each species belong to, color of bar is consistent with the species’ color.

We reconstructed separate phylogenetic trees based on different methods: Bayesian and ML analyses on CCG. Bayesian and ML analyses recovered almost identical trees from each data set. There was strong support for the monophyly of each family were revealed based on CCG data (Figure 7). The topological structures from the SCG and CDS are similar to that from CCG, and all lineages possess high bootstrap values (Supplementary Figure S3). *Agapanthus coddii* from Agapanthoideae had strong support to be a sister to the Allioideae, and Asparagaceae was supported to be the sister of Agapanthoideae and Allioideae. For Chinese species in section *Daghestanica*, *A. maowenense* was closely clustered with *A. herderianum*, and *A. chrysanthum* is sister to the *A. rude*. All six species were closely clustered in one lineage (Figure 7 and Supplementary Figure S3).

### Positive Selection Analyses

**FIGURE 8:**
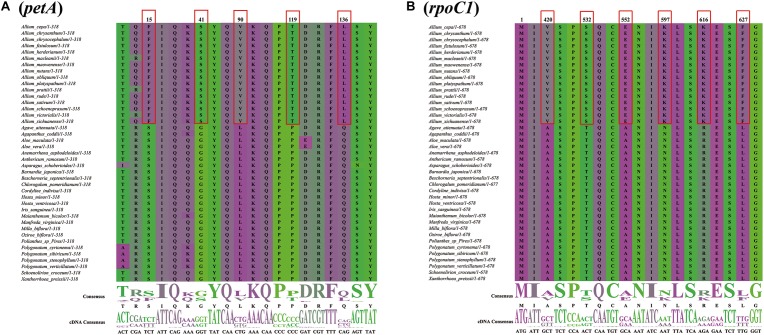
Partial alignment of two out of 10 positively selected genes. **(A)** Partial aligned amino acids sequences of the *petA* gene; **(B)** partial aligned amino acids sequences of the *rpoC1* gene. The red blocks stand for the amino acids in *Allium* (Allioideae) with a high BEB posterior probability.

There were 50 single-copy CDS genes initially considered for the positive selection analysis (Supplementary Table S9), but 44 were eventually selected after filtering (Table 2). All *p*-values were not significant in each gene range, however, ten protein coding genes (*petA*, *psbD*, *psbE*, *ycf3*, *psaI*, *rps4*, *psbM*, *ndhE*, *ndhG*, and *rpoC1*) were found with significant posterior probabilities suggesting sites with positive selection in the BEB test. Among them, most genes only had one positive selective site, whereas *rpoC1* gene possessed six positive selective sites, followed by *petA* and *ndhE* that had five and three positive selective sites, respectively (Figure 8, Supplementary Figure S4, and Table 2).

**Table 2:** The potential positive selection test based on the branch-site model.

Gene	Null hypothesis			Alternative hypothesis			Significance test	
	
name	lnL	df	omega (*w* = 1)	lnL	df	omega (*w* > 1)	BEB	*P*-value
*rpl36*	-321.922186	83	1	-321.96393	84	1	BEB:	7.73E-01
*ndhH*	-4212.542434	83	1	-4212.542469	84	1	BEB:	9.93E-01
*ycf4*	-1778.248142	83	1	-1778.248143	84	1	BEB:	9.99E-01
***petA***	-2906.976569	83	1	-2906.927721	84	3.9584	BEB:15, S, 0.611; 41, G, 0.612; 90, L, 0.591; 119, P, 0.601; 136, Q, 0.609	7.55E-01
***psbD***	-2586.660506	83	1	-2586.660506	84	1	BEB:8, F, 0.510	1.00E+00
***psbE***	-521.890656	83	1	-521.890656	84	1	BEB:11, A, 0.637	1.00E+00
*rps11*	-1493.350251	83	1	-1493.350251	84	1	BEB:	1.00E+00
***ycf3***	-1386.13585	83	1	-1386.221167	84	1	BEB:136, F, 0.560	6.80E-01
*psbL*	-208.400691	83	1	-208.400698	84	2.87887	BEB:	9.97E-01
*ndhC*	-1077.629435	83	1	-1077.629435	84	1	BEB:	1.00E+00
*atpA*	-4966.799781	83	1	-4966.799781	84	1	BEB:	1.00E+00
*psaA*	-5689.34135	83	1	-5689.341353	84	1	BEB:	9.98E-01
*atpI*	-2008.387924	83	1	-2008.387924	84	1	BEB:	1.00E+00
*psaC*	-607.584019	83	1	-607.584019	84	1	BEB:	1.00E+00
*rps8*	-1256.138747	83	1	-1256.138622	84	1	BEB:	9.87E-01
*rpl14*	-1026.273839	83	1	-1026.273839	84	1	BEB:	1.00E+00
*rps3*	-2622.32359	83	1	-2622.32359	84	1	BEB:	1.00E+00
*psaB*	-5586.326789	83	1	-5586.326796	84	1	BEB:	9.97E-01
*rps15*	-1308.749535	83	1	-1308.749535	84	1	BEB:	1.00E+00
*rps14*	-874.066639	83	1	-874.066639	84	1	BEB:	1.00E+00
*psbH*	-661.970559	83	1	-661.970557	84	1	BEB:	9.98E-01
***psaI***	-316.38643	83	1	-315.817422	84	1	BEB:9, I, 0.606	2.86E-01
*atpH*	-527.187559	83	1	-527.187499	84	1	BEB:	9.91E-01
*petG*	-250.570589	83	1	-250.570589	84	1	BEB:	1.00E+00
***rps4***	-1822.237453	83	1	-1822.237453	84	1	BEB:159, G, 0.558	1.00E+00
*petB*	-1731.777196	83	1	-1731.777196	84	1	BEB:	1.00E+00
***psbM***	-256.645394	83	1	-256.086734	84	1	BEB:17, V, 0.856	2.91E-01
*rps18*	-905.190682	83	1	-905.190682	84	1	BEB:	1.00E+00
***ndhE***	-1052.1543	83	1	-1051.275166	84	9.32678	BEB:47, L, 0.803; 97, S, 0.667; 100, K, 0.526	1.85E-01
*psbK*	-657.035777	83	1	-657.035777	84	1	BEB:	1.00E+00
***ndhG***	-2065.495882	83	1	-2065.495882	84	1	BEB:81, V, 0.547	1.00E+00
*psbA*	-2688.234591	83	1	-2688.234591	84	1	BEB:	1.00E+00
*psbF*	-251.623828	83	1	-251.623828	84	2.83214	BEB:	1.00E+00
*rpoB*	-9973.949393	83	1	-9973.949395	84	1	BEB:	9.98E-01
*rpl16*	-1601.948795	83	1	-1601.948795	84	1	BEB:	1.00E+00
*rpl33*	-602.061616	83	1	-602.061663	84	1	BEB:	9.92E-01
*psbB*	-4343.009364	83	1	-4343.009362	84	1	BEB:	9.98E-01
*psbC*	-3577.97404	83	1	-3577.974038	84	1	BEB:	9.98E-01
*petN*	-177.723478	83	1	-177.723478	84	1	BEB:	1.00E+00
***rpoC1***	-6352.847044	83	1	-6352.847006	84	1	BEB:420, A, 0.561; 532, T, 0.588; 552, A, 0.584; 597, N, 0.561; 616, R, 0.558; 627, L, 0.517	9.93E-01
*petL*	-237.316155	83	1	-237.316155	84	1	BEB:	1.00E+00
*psbN*	-330.808775	83	1	-333.305277	84	1	BEB:	2.55E-01
*psbT*	-237.823263	83	1	-237.823263	84	1	BEB:	1.00E+00
*psbJ*	-288.795385	83	1	-288.795378	84	1	BEB:	9.97E-01
*ndhJ*	-1408.504592	83	1	-1408.504592	84	1	BEB:	1.00E+00


*Bold types are genes with positively selected sites. BEB, Bayesian Empirical Bayes.*

## Discussion

### Sequence Differentiation of Chinese Species in *Allium* Section *Daghestanica*

Recently, chloroplast genomes have been used to evaluate the genetic divergence among related species (Bellusci et al., 2008; Song et al., 2015; Xie et al., 2018). Comparative genome analysis of the six Chinese *Allium* section *Daghestanica* species showed highly conserved structures, which can be inferred from the similar gene number, gene component, genome size and the types of simple sequence repeats (SSRs) (Table 1, Supplementary Tables S4, S8, and Supplementary Figure S1). Due to the high polymorphic rate, SSRs have been recognized as one of the main sources of molecular markers and have been extensively researched in phylogenetic and biogeographic studies of populations (Powell et al., 1995; Provan et al., 1997; Pauwels et al., 2012). Six genes (*trnK-UUU*, *matK*, *trnG-UCC*, *trnG-GCC*, *ndhF* and *rps15*) with nucleotide diversity more than 0.0700 and five genes (*accD*, *clpP*, *rpl16*, *ccsA*, and *ndhA*) with nucleotide diversity more than 0.05500 were detected (Supplementary Figure S2). Among these loci, *clpP*, *accD*, *ndhF*, *rps15*, and *ccsA* have been previously detected as highly variable regions in different plants (Kim and Lee, 2005; Dong et al., 2012; Qian et al., 2013; Hu et al., 2016). We believe that these SSRs and genes with high nucleotide diversity are good sources for interspecies phylogenetic analysis in the future.

### The Phylogenetic Analysis of Chinese Species in Section *Daghestanica*

The results of our phylogenetic analysis strongly support that *Allium* is monophyletic, which is in accordance with previous studies (Friesen et al., 2006; Nguyen et al., 2008; Li et al., 2010). We also found that *Agapanthus coddii* was closely related to *Allium* (Allioideae) (Figure 7 and Supplementary Figure S3). The relationships of Chinese species in section *Daghestanica* were resolved: the position of *A. herderianum* was confirmed, which showed a close relationship with *A. maowenense*, and differentiated early. *A. chrysanthum* was tightly clustered with *A. rude*, which is inconsistent with Li et al. (2010), who showed that *A. chrysanthum* was closely related to *A. xichuanense*, and *A. rude* was clustered with *A. chrysocephalum*. Our results may be more reliable, since we used the whole chloroplast genome (CCG) (Figure 7), SCG and CDS data (Supplementary Figure S3) respectively in phylogeny reconstruction compared to the *rps16* fragment in study of Li et al. (2010), and our results were also supported by the morphological characteristics in Yu et al. (2018), which showed similar testa cells of *A. chrysanthum* and *A. rude* that are not parallel and irregular in long axis, and their filaments are longer than perianth segments (Figure 1). Although the *A. herderianum* has a close relationship with *A. maowenense*, the morphological characteristics of them are distinct different, and *A. maowenense* is most differentiated in morphology among the six species in terms of its perianth color, green midrib in perianth (Figure 1, 7). In addition, Yu et al. (2018) found that the testa cells of *A. maowenense* are parallel and irregular, which are obviously different from the other five species. The outer perianth segments of *A. chrysocephalum* and *A. herderianum* are boat-shaped, and their style are longer than perianths, these characteristics are distinct from the other four species (Figure 1). The leaves of *A. chrysocephalum* are flat falcate and in *A. herderianum* are semiterete and fistulose, easily differentiating the two species. However, we did not find a close relationship between *A. chrysocephalum* and *A. herderianum* (Figure 7). Therefore, our study uncovered a new relationship of Chinese species in *Allium* section *Daghestanica* using the whole cp genome data.

### The Adaptation Evolution of *Allium* (Allioideae) Plastome

A previous study using 150 species cp genomes showed that the GC content of these species ranged from 19.5 to 42.1% (Smith, 2009). We found that Allioideae species typically exhibited lower GC content than other non-Allioideae families’ species (Figure 3). There are two reasons that may result in lower GC content of plastid DNA, firstly, a neutral mutation process such as AT-mutation pressure or AT-biased gene conversion, which will reduce the GC content (Howe et al., 2003; Kusumi and Tachida, 2005; Khakhlova and Bock, 2010), and secondly, that selection for translational efficiency may lead to the lack of G and C observed in plastid genomes (Morton, 1993, 1998; And and Voelker, 2003). We suggest that mutation pressure from the evolution process may be a crucial factor resulting in the low GC content of Allioideae. The Ka/Ks ratios of Allioideae species are exceptionally high compared with those observed within non-Allioideae families’ species and this may be an indication of an elevated mutation rate in Allioideae plastid DNA (Figure 4). Mutations during evolutionary processes leading to reduced GC content has also been found in mitochondrial DNA, nucleomorph DNA, and in the genomes of symbionts, parasites, and pathogenic bacteria (Ogata et al., 2001; And and Voelker, 2003; Lane et al., 2007; Smith and Lee, 2008).

Elevated pairwise Ka/Ks ratios were observed in Allioideae species pairs compared to non-Allioideae species pairs (Figure 4 and Supplementary Table S7). The elevated Ka/Ks ratios are unlikely to be explained by changes in codon preference since we did not obtain obvious codon usage bias in Allioideae species (Figure 5). It is possible that other factors (e.g., habitat environment and adaptation evolution) may have contributed to the elevated Ka/Ks ratios. Allioideae is a variable group that is spread widely across the Holarctic region from the dry subtropics to the boreal zone (Friesen et al., 2006; Li et al., 2010). Furthermore, species in Allioideae grow in various conditions from dry and well-drained soils to moist and organic soils, with most growing in sunny locations, and a number of species also grow in forests, or even in swamps or water (Block, 2010); The environment imposes stressful living conditions on Allioideae species and results in species divergence. The Ka/Ks ratios have been widely used to infer the evolutionary dynamics and identify adaptive signatures among species (Yang and Bielawski, 2000; Fay and Wu, 2003; Ai et al., 2015), and elevated Ka/Ks ratios indicate species may have undergone more selective forces (Hurst, 2002). Thus, the elevated Ka/Ks ratios observed throughout the Allioideae may suggest species in Allioideae undergo some selection pressure that is unknown.

Gene losses and gains are considered as important adaptive processes that greatly contribute to trait evolution (Hahn et al., 2007; Ding et al., 2012). In this study, we found that the gene *rps2* was lost in all *Allium* (Allioideae) species, with *infA*, *rps16*, *psbZ*, *ndhD*, *cemA*, *rps19*, *ycf1*, and *rpl32* were lost in some species of Allioideae, Asparagaceae and Asphodeloideae (Figure 6). Gene *infA*, which codes for translation initiation factor 1, was lost in an early ancestor of Fabales and Cucurbitales (Millen et al., 2001), and it was found as pseudogene in many genus (e.g., *Albuca*, *Behnia*, *Camassia*, and *Echeandia*) in study of McKain et al. (2016). The study of Steele et al. (2012) identified that gene *rps16*, *rpl32*, and *rps19* were missing from various taxa throughout Asparagales, and these shared losses were suggested as the result of common ancestry. The gene *rps2* was also identified as a pseudogene in *Chlorophytum rhizopendulum* (McKain et al., 2016). However, the mechanism underlying the loss of the *rps2* gene in Allioideae was poorly understood. A previous study indicated that the product of the *rps2* gene plays an important role in defense signal transduction (Bent et al., 1994). Although we detected the loss of the *rps2* gene in Allioideae, we did not find a cause. Therefore, further studies are needed to examine whether specific factors were associated with the loss of the *rps2* genes in the Allioideae.

### Positive Selection of *Allium* (Allioideae) Plastome

We investigated PSGs to detect genes in the Allioideae lineage that may have evolved to adapt to environmental conditions. Ten genes with significant posterior probabilities for codon sites were identified in the BEB test, although the positive selection was not significant in all genes (*p*-value > 0.05) (Figure 8, Supplementary Figure S4, and Table 2). Yang et al. (2005) suggested that codon sites with higher posterior probability can be regarded as positively selected sites, and genes that possessing the positively selected sites may be evolving under divergent selective pressures, which indicate that these ten genes may be under positive selection pressure. Notably, we found that seven of these ten genes are associated with photosystem I and II subunits (*psbD*, *psbE*, *psbM*, and *psaI*), NADH-dehydrogenase subunits (*ndhE* and *ndhG*) and subunits of cytochrome b/f complex (*petA*) (Table 2). Photosystem I and II are sites of the photosynthetic light reactions of plants (Golbeck, 1987), and are integral membrane protein complexes that use light energy to produce the high energy carriers ATP and NADPH (Weiss et al., 1991; Yamori and Shikanai, 2016). NADH-dehydrogenase subunits and cytochrome b/f complex are essential in the electron transport chain for generation of ATP (Weiss et al., 1991; Cramer et al., 2011; Xiao et al., 2012), and are all important components for photosynthesis of plants. Therefore, all these genes are indispensable components for photosynthesis, which is the most important process for plant growth and development (Bryant and Frigaard, 2006). Among all PSGs, we found that the *rpoC1* gene possessed the maximum number of sites under positive selection in Allioideae species (Figure 8, Table 2, and Supplementary Figure S4). This suggests that the *rpoC1* gene may play a pivotal role in the adaptive evolution of Allioideae species. We also observed site-specific selection in *rps4* gene that has important role in the chloroplast ribosome (Rogalski et al., 2006; Tiller and Bock, 2014). Most of genes mentioned above have been reported under positive selection in previous studies (Dong et al., 2018; Fan et al., 2018; Wu et al., 2018). Species in Allioideae are mostly characterized by tunicated bulbs and narrow basal leaves (Li et al., 2010), and these are key traits that likely contributed to their adaptation to diverse harsh environments, and generated and maintained high levels of plant diversity. The results of high Ka/Ks ratios also suggested positive selection existed in Allioideae species (Figure 4 and Supplementary Table S7). Consequently, most PSGs may have played key roles in the adaptation of species in the Allioideae during the evolution process.

## Author Contributions

D-FX, YY, X-JH, and Y-QD conceived and designed the experiments. D-FX, Y-QD, YY, and H-XY analyzed the sequence data and drafted the manuscript. D-FX, S-DZ, H-XY, CX, and J-PC participated in data analysis and manuscript drafting. D-FX, MP, X-JH, and YY revised the manuscript. All authors read and approved the final manuscript.

## Conflict of Interest Statement

The authors declare that the research was conducted in the absence of any commercial or financial relationships that could be construed as a potential conflict of interest.

## Footnotes

1 http://codonw.sourceforge.net

## Supplementary Material

Figure S1 Analysis of simple sequence repeats (SSRs) in chloroplast genomes of Section *Daghestanica* species. Number of different SSR types detected in each species.

Figure S2 The nucleotide diversity of the whole chloroplast genomes of Section *Daghestanica* species. LSC, large single-copy region; IRs, inverted repeats region; SSC, small single-copy region.

Figure S3 Phylogenetic tree reconstruction of 41 taxa based on **(A)** the single copy gene sequences and **(B)** the CDS sequences. Numbers above the branches are the posterior probabilities of BI and the bootstrap values of ML, respectively.

Figure S4 Partial alignment of amino acids sequences in another eight positively selected genes. **(A–H)**: *psaI*, *psbD*, *psbE*, *psbM*, *ndhE*, and *ndhG*, *ycf3* and *rps4*. The red blocks stand for the amino acids in Allioideae with a high BEB posterior probability.

Table S1 Information for sample collection.

Table S2 Primers used for gap closure in this study.

Table S3 The Genbank accessions of all 41 taxa cp genome sequences used this study.

Table S4 Gene content in Section *Daghestanica* species cp genomes. ^∗^Genes with two copies.

Table S5 Summary of complete chloroplast genomes of all 41 taxa used in this study.

Table S6 The statistics of codon usage bisa in all 41 taxa used in this study.

Table S7 Summary of Pairwise Ka/Ks ratios in *Allium* (Allioideae) and other families.

Table S8 Statistics of simple sequence repeats in each species of Section *Daghestanica*.

Table S9 List of single-copy genes extracted from cp genome.
