# Supplementary material for: Phylogeny of Chinese Allium Species in Section Daghestanica and Adaptive Evolution of Allium (Amaryllidaceae, Allioideae) Species Revealed by the Chloroplast Complete Genome
Source: Front Plant Sci. 2019 Apr 30;10:460. doi: 10.3389/fpls.2019.00460 (PMC6503222; doi:10.3389/fpls.2019.00460)
Supplement: Figure S1 — Analysis of simple sequence repeats (SSRs) in chloroplast genomes of Section Daghestanica species. Number of different SSR types detected in each species. [file Data_Sheet_1.zip › Supplementary Table S1.doc]

**Table S1 Information for sample collections.**

| **Species** | **Sampling locality** | **latitude**  **(N)** | **Longitude**  **(E)** | **Alt. (m)** |
| --- | --- | --- | --- | --- |
| ***Allium chrysanthum*** | Luqu county, Gansu | 34.51748° | 102.77266° | 3165 |
| ***Allium rude*** | Xiahe county, Gansu | 35.18834° | 102.53379° | 3071 |
| ***Allium xichuanense*** | Kangding county, Sichuan | 30.28045° | 101.84195° | 3847 |
| ***Allium chrysocephalum*** | Xiahe county, Gansu | 35.16169° | 102.57590° | 3266 |
| ***Allium maowenense*** | Mao county, Sichuan | 31.67487° | 103.82124° | 1478 |
| ***Allium herderianum*** | Kangle, Gansu | 34.93995° | 103.76051° | 3161 |
