# Supplementary material for: Phylogeny of Chinese Allium Species in Section Daghestanica and Adaptive Evolution of Allium (Amaryllidaceae, Allioideae) Species Revealed by the Chloroplast Complete Genome
Source: Front Plant Sci. 2019 Apr 30;10:460. doi: 10.3389/fpls.2019.00460 (PMC6503222; doi:10.3389/fpls.2019.00460)
Supplement: Figure S1 — Analysis of simple sequence repeats (SSRs) in chloroplast genomes of Section Daghestanica species. Number of different SSR types detected in each species. [file Data_Sheet_1.zip › Supplementary Table S2.doc]

**Table S2 Primers used for gap closure in this study.**

| **Primer** | **Direction** | **Sequence** | **Tm (℃)** |
| --- | --- | --- | --- |
| 1 | F | GTTTCTCCTCATACGGCTCG | 57.5 |
|  | R | TGCACGCTGAACTTCCTG |  |
| 2 | F | GTGCTAATGCCACAACGA | 53.5 |
|  | R | TAGGCCCAGAATGGTTTT |  |
| 3 | F | CAAATCGGCTCGTTCTGC | 56.5 |
|  | R | GCAACCAGGAAGCGAAAG |  |
| 4 | F | AAGAACCGATCCCAACGA | 55.6 |
|  | R | TAGGGTATGTTTAGCAAATGGA |  |
| 5 | F | CACGGGCAAAGTAAGGGAA | 58.5 |
|  | R | AGCCTGCTGCTGAGTGGTTTGC |  |
| 6 | F | GAACCGCTAGACGATGGG | 55.8 |
|  | R | GAGGCATTGGTCAGTAGGTATT |  |

The PCR program began with 4-min initial denaturing at 94 ºС followed by 35 cycles of 1-min denaturation at 94 ºС, 1-min annealing at abovementioned Tm, and 1.5-min extension at 72 ºС, a final extension was run for 5 min at 72 ºС.
