# Supplementary material for: Phylogeny of Chinese Allium Species in Section Daghestanica and Adaptive Evolution of Allium (Amaryllidaceae, Allioideae) Species Revealed by the Chloroplast Complete Genome
Source: Front Plant Sci. 2019 Apr 30;10:460. doi: 10.3389/fpls.2019.00460 (PMC6503222; doi:10.3389/fpls.2019.00460)
Supplement: Figure S1 — Analysis of simple sequence repeats (SSRs) in chloroplast genomes of Section Daghestanica species. Number of different SSR types detected in each species. [file Data_Sheet_1.zip › Supplementary Table S3.DOC]

Table S3 The Genbank accessions of all 41 taxa cp genome sequences used this study.

| **Species** | **NCBI accessions** |
| --- | --- |
| *Polianthes sp Pires* | KX931464 |
| *Manfreda virginica* | NC_032707 |
| *Agave attenuata* | NC_032696 |
| *Beschorneria septentriona* | NC_032699 |
| *Chlorogalum pomeridianum* | NC_032701 |
| *Schoenolirion croceum* | NC_032710 |
| *Hosta ventricosa* | NC_032706 |
| *Hosta minor* | NC_035999 |
| *Anthericum ramosum* | NC_035972 |
| *Anemarrhena asphodeloides* | NC_032698 |
| *Barnardia japonica* | NC_035997 |
| *Oziroe biflora* | KX931463 |
| *Milla biflora* | NC_036000 |
| *Cordyline indivisa* | NC_035998 |
| *Asparagus schoberioides* | NC_035969 |
| *Polygonatum verticillatum* | NC_028523 |
| *Polygonatum sibiricum* | NC_029485 |
| *Polygonatum cyrtonema* | NC_028429 |
| *Polygonatum stenophyllum* | NC_035995 |
| *Maianthemum bicolor* | NC_035970 |
| ***Allium chrysanthum*** | MH992108 available at: <https://www.ncbi.nlm.nih.gov/nuccore/MH992108> |
| ***Allium rude*** | MH992112 available at: <https://www.ncbi.nlm.nih.gov/nuccore/MH992112> |
| ***Allium xichuanense*** | MH992113 available at: <https://www.ncbi.nlm.nih.gov/nuccore/MH992113> |
| ***Allium chrysocephalum*** | MH992109 available at: <https://www.ncbi.nlm.nih.gov/nuccore/MH992109> |
| ***Allium maowenense*** | MH992111 available at: <https://www.ncbi.nlm.nih.gov/nuccore/MH992111> |
| ***Allium herderianum*** | MH992110 available at: <https://www.ncbi.nlm.nih.gov/nuccore/MH992110> |
| ***Allium schoenoprasum*** | LT699700 |
| *Allium fistulosum* | LT674586 |
| *Allium cepa* | KM088014 |
| *Allium platyspathum* | LT673892 |
| *Allium obliquum* | LT699701 |
| *Allium nutans* | LT799837 |
| *Allium sativum* | KY085913 |
| *Allium macleanii* | LT699703 |
| *Allium victorialis* | NC_037240 |
| *Allium prattii* | MG739457 |
| *Agapanthus coddii* | NC_035971 |
| *Aloe vera* | KX377524 |
| *Aloe maculata* | KX377523 |
| *Xanthorrhoea preissii* | NC_035996 |
| *Iris sanguinea* | NC_029227 |
