# Supplementary material for: Phylogeny of Chinese Allium Species in Section Daghestanica and Adaptive Evolution of Allium (Amaryllidaceae, Allioideae) Species Revealed by the Chloroplast Complete Genome
Source: Front Plant Sci. 2019 Apr 30;10:460. doi: 10.3389/fpls.2019.00460 (PMC6503222; doi:10.3389/fpls.2019.00460)
Supplement: Figure S1 — Analysis of simple sequence repeats (SSRs) in chloroplast genomes of Section Daghestanica species. Number of different SSR types detected in each species. [file Data_Sheet_1.zip › Supplementary Table S4.doc]

**Table S4 Gene content in Section *Daghestanica* species cp genomes.**

| **Category for genes** | **Group of genes** | **Name of genes** |
| --- | --- | --- |
| **Self-replication** | transfer RNAs | trnA-UGC*, trnC-GCA, trnD-GUC, trnE-UUC, trnF-GAA, trnfM-CAU, trnG-GCC, trnG-UCC, trnI-CAU*, trnI-GAU*, trnK-UUU, trnL-CAA*, trnL-UAA, trnL-UAG, trnM-CAU, trnN-GUU*, trnP-UGG, trnQ-UUG, trnR-ACG*, trnR-UCU, trnS-GCU, trnS-GGA, trnS-UGA, trnT-GGU, trnT-UGU, trnV-GAC*, trnV-UAC, trnW-CCA, trnY-GUA, trnH-GUG* |
|  | ribosomal RNAs | rrn4.5*, rrna5*, rrn16*, rrn23* |
|  | RNA polymerase | rpoA, rpoB, rpoC1, rpoC2 |
|  | Small subunit of ribosomal proteins (SSU) | rps3, rps4, rps7*, rps8, rps11, rps12*, rps14, rps15, rps16, rps18, rps19* |
|  | Large subunit of ribosomal proteins (LSU) | rpl2*, rpl14, rpl16, rpl20, rpl22, rpl23*, rpl32, rpl33, rpl36 |
| **Genes for photosynthesis** | Subunits of NADH-dehydrogenase | ndhA, ndhB*, ndhC, ndhD, ndhE, ndhF, ndhG, ndhH, ndhI, ndhJ, ndhK |
|  | Subunits of photosystem I | psaA, psaB, psaC, psaI, psaJ |
|  | Subunits of photosystem II | psbA, psbB, psbC, psbD, psbE, psbF, psbH, psbI, psbJ, psbK, psbL, psbM, psbN, psbT, psbZ |
|  | Subunits of cytochrome b/f complex | petA, petB, petD, petG, petL, petN |
|  | Subunits of ATP synthase | atpA, atpB, atpE, atpF, atpH, atpI |
|  | Large subunit of rubisco | rbcL |
| **Other genes** | Tanskational initiation factor | infA |
|  | Protease | clpP |
|  | Maturase | matK |
|  | Subunit of Acetyl-CoA-carboxylase | accD |
|  | Envelope membrane protein | cemA |
|  | C-type cytochrome synthesis gene | ccsA |
| **Genes of unknown function** | hypothetical chloroplast reading frames (ycf) | ycf1*, ycf2*, ycf3, ycf4 |

***Gene with two copies**
