# Supplementary material for: Phylogeny of Chinese Allium Species in Section Daghestanica and Adaptive Evolution of Allium (Amaryllidaceae, Allioideae) Species Revealed by the Chloroplast Complete Genome
Source: Front Plant Sci. 2019 Apr 30;10:460. doi: 10.3389/fpls.2019.00460 (PMC6503222; doi:10.3389/fpls.2019.00460)
Supplement: Figure S1 — Analysis of simple sequence repeats (SSRs) in chloroplast genomes of Section Daghestanica species. Number of different SSR types detected in each species. [file Data_Sheet_1.zip › Table 5.doc]

**Table S5 Summary of complete chloroplast genomes of all 41 taxa used in this study.**

| Taxon | Full | | Gene Number | Protein-coding | tRNAs | rRNAs |
| --- | --- | --- | --- | --- | --- | --- |
|  | length (bp) | GC (%) |  |  |  |  |
| *Polianthes sp Pires* | 157,337 | 37.8 | 124 | 86 | 38 | 8 |
| *Manfreda virginica* | 157,308 | 37.8 | 132 | 86 | 38 | 8 |
| *Agave attenuata* | 157,451 | 37.8 | 132 | 86 | 38 | 8 |
| *Beschorneria septentrionalis* | 157,043 | 37.8 | 132 | 85 | 38 | 8 |
| *Chlorogalum pomeridianum* | 157,288 | 37.7 | 131 | 84 | 38 | 8 |
| *Schoenolirion croceum* | 156,605 | 37.9 | 132 | 84 | 38 | 8 |
| *Hosta ventricosa* | 156,577 | 37.8 | 132 | 82 | 38 | 8 |
| *Hosta minor* | 156,674 | 37.8 | 132 | 84 | 38 | 8 |
| *Anthericum ramosum* | 155,812 | 37.3 | 133 | 82 | 38 | 8 |
| *Anemarrhena asphodeloides* | 156,867 | 37.8 | 132 | 86 | 38 | 8 |
| *Barnardia japonica* | 156,079 | 37.7 | 133 | 84 | 38 | 8 |
| *Oziroe biflora* | 155,633 | 37.7 | 124 | 86 | 38 | 8 |
| *Milla biflora* | 156,684 | 37.6 | 133 | 85 | 38 | 8 |
| *Cordyline indivisa* | 155,196 | 37.8 | 131 | 84 | 37 | 8 |
| *Asparagus schoberioides* | 156,875 | 37.6 | 132 | 86 | 37 | 8 |
| *Polygonatum verticillatum* | 154,639 | 37.8 | 136 | 84 | 38 | 8 |
| *Polygonatum sibiricum* | 152,960 | 37.9 | 136 | 84 | 38 | 8 |
| *Polygonatum cyrtonema* | 153,503 | 37.3 | 136 | 83 | 38 | 8 |
| *Polygonatum stenophyllum* | 156,028 | 37.8 | 133 | 85 | 38 | 8 |
| *Maianthemum bicolor* | 157,176 | 37.7 | 133 | 86 | 38 | 8 |
| *Allium chrysanthum* | 153,621 | 36.8 | 132 | 86 | 38 | 8 |
| *Allium rude* | 153,697 | 36.7 | 132 | 86 | 38 | 8 |
| *Allium xichuanense* | 153,673 | 36.7 | 132 | 86 | 38 | 8 |
| *Allium chrysocephalum* | 153,710 | 36.8 | 132 | 86 | 38 | 8 |
| *Allium maowense* | 153,608 | 36.8 | 132 | 86 | 38 | 8 |
| *Allium herderianum* | 153,605 | 36.8 | 132 | 86 | 38 | 8 |
| *Allium schoenoprasum* | 153,014 | 36.8 | 132 | 86 | 38 | 8 |
| *Allium fistulosum* | 152,859 | 36.9 | 132 | 86 | 38 | 8 |
| *Allium cepa* | 153,568 | 36.8 | 132 | 86 | 38 | 8 |
| *Allium platyspathum* | 152,529 | 36.8 | 131 | 85 | 38 | 8 |
| *Allium obliquum* | 152,597 | 36.8 | 132 | 86 | 38 | 8 |
| *Allium nutans* | 153,456 | 36.9 | 132 | 86 | 38 | 8 |
| *Allium sativum* | 153,118 | 36.7 | 134 | 83 | 37 | 8 |
| *Allium macleanii* | 152,633 | 36.9 | 131 | 85 | 38 | 8 |
| *Allium victorialis* | 154,074 | 37.0 | 132 | 85 | 38 | 8 |
| *Allium prattii* | 154,482 | 37.0 | 131 | 85 | 38 | 8 |
| *Agapanthus coddii* | 157,055 | 37.5 | 133 | 86 | 38 | 8 |
| *Aloe vera* | 152,875 | 37.7 | 131 | 85 | 38 | 8 |
| *Aloe maculata* | 153,175 | 37.6 | 131 | 85 | 38 | 8 |
| *Xanthorrhoea preissii* | 158,116 | 37.9 | 133 | 86 | 38 | 8 |
| *Iris sanguinea* | 152,408 | 38.0 | 133 | 87 | 38 | 8 |
